# Enhancing the Visibility of Delamination during Pulsed Thermography of Carbon Fiber-Reinforced Plates Using a Stacked Autoencoder

**DOI:** 10.3390/s18092809

**Published:** 2018-08-25

**Authors:** Changhang Xu, Jing Xie, Changwei Wu, Lemei Gao, Guoming Chen, Gangbing Song

**Affiliations:** 1 College of Mechanical and Electronic Engineering, China University of Petroleum, Qingdao 266580, China; xiejing@upc.edu.cn (J.X.); 18754280118@163.com (C.W.); gaolemeihx@163.com (L.G.); gmchen@upc.edu.cn (G.C.); 2 Department of Mechanical Engineering, University of Houston, Houston, TX 77004, USA

**Keywords:** stacked autoencoder (SAE), pulsed thermography (PE), delamination detection, carbon fiber-reinforced polymer

## Abstract

The effectiveness of pulsed thermography (PT) for detecting delamination in carbon fiber-reinforced polymer (CFRP) plates has been widely verified. However, delaminations are usually characterized by weak visibility due to the influences of inspection factors and the delaminations with weak visibility are easily missed in real inspections. In this study, by introducing a deep learning algorithm—stacked autoencoder (SAE)—to PT, we propose a novel approach (SAE-PT) to enhance the visibility of delaminations. Based on the ability of SAE to learn unsupervised features from data, the thermal features of delaminations are extracted from the raw thermograms. The extracted features are then employed to construct SAE images, in which the visibility of delaminations is expected to be enhanced. To test the performance of SAE-PT, we inspected CFRP plates with prefabricated delaminations. By implementing SAE-PT on the raw inspection data, the delaminations were more clearly indicated in the constructed SAE images. We also compare SAE-PT to the widely used principal component thermography (PCT) method to further verify the validity of the proposed approach. The results reveal that compared to PCT, SAE-PT can show delaminations in CFRP with higher contrast. By effectively enhancing the delamination visibility, SAE-PT thus has potential for improving the inspection accuracy of PT for non-destructive testing (NDT) of CFRP.

## 1. Introduction

Carbon fiber-reinforced polymer (CFRP) is extensively applied in structural engineering due to its high strength to weight ratio, high stiffness and powerful corrosion resistance [1,2,3,4]. However, internal and invisible defects can significantly deteriorate the residual stiffness and load-bearing capacity of CFRP [5,6,7,8], hence detecting such defects of CFRP is critical to guarantee the safety of CFRP structures. A delamination, which is usually the result of an impact, is one typical kind of internal and invisible defect seen in CFRP. With the recent emphasis on structural health monitoring and damage detection, detection of delamination is an area that attracts intense research interest.

Pulsed thermography (PT), which is an important non-destructive testing (NDT) technique, has been verified effective for detecting delamination in CFRP [9,10,11]. However, the delamination visibility during PT is significantly influenced by some factors, including the performance of the infrared imager, the dimension and location of a delamination, and the quantity of heat energy injected into the inspected CFRP [12,13,14,15]. As a result, delaminations generally appear with low visibility in the raw thermograms, which significantly decreases the detection accuracy of PT and may even result in a false inspection result. Therefore, enhancing delamination visibility is critical to improve the detection accuracy of PT for delaminations in CFRP.

Benefitting from the development of signal processing algorithms, many approaches have been developed to improve defect visibility during PT inspections. The following approaches have gained wide applications in a variety of fields: thermal signal reconstruction (TSR), pulsed phase thermography (PPT) and principle component thermography (PCT). TSR employs a standard polynomial fitting to approximate the temperature variations of the inspection surface during PT based on the heat conduction theory [16]. The images reconstructed with the fitting data, or with the first and the second derivatives of the fitting data usually show the defects indications in a more distinct manner. PPT implements discrete Fourier transform (DFT) on each pixel, and then the amplitude and the phase information are used to construct new images [17]. As a result, clearer defect indications can usually be obtained in the constructed images. Combining principle component analysis (PCA) with PT [18,19], PCT processes the temperature variation of each pixel by using PCA and thereby the calculated loads are used to construct new images. Commonly, the images corresponding to the first few principal components indicate the defects with enhancing visibility. The success of these methods verifies the powerful capability of signal processing techniques to enhance the defect visibility and to improve the detection accuracy during PT.

In recent years, deep learning (DL) algorithms have attracted extensive interest owing to their outstanding performances in machine learning. Various DL algorithms have been employed in a variety of applications, such as image recognition [20,21,22,23], speech recognition [24,25,26] and language understanding [27], defect detection [28], and target recognition [29]. However, little attention has been paid to introducing DL to infrared thermography (IRT) to detect defects in materials. In our previous study, we have verified the capability of autoencoder (AE) to improve the visibility of rear cracks of metal plates during inductive thermography [30]. As an important DL algorithm, a stacked autoencoder (SAE) is constructed with a few AEs. It usually presents better performance than other classical methods in solving machine learning problems [31,32]. Its excellent performance in other applications inspires us to study its potential to improve the inspection results of PT.

Based on our previous study on introducing AE into inductive thermography to detect rear cracks in metal plates [30], here we further investigate the effectiveness of SAE to improve the inspection capability of PT for CFRP. A novel approach named SAE-PT is proposed to enhance the visibility of delaminations during PT of CFRP. We validate the efficacy of the proposed approach through PT inspection on two CFRP plates with prefabricated delaminations. We visually compared the raw thermograms, the EOFs obtained through PCT and the SAE images of SAE-PT. We also quantitatively compared the delamination visibility in these images using defect contrast as the metric.

The rest of this paper is organized as follows: Section 2 introduces the background knowledge about SAE and describes the proposed SAE-PT methodology in detail. Experimental investigation is presented in Section 3 and the experimental results are discussed with a comparison between SAE-PT and PCT in Section 4. Finally, Section 5 concludes this work.

## 2. Methodology

### 2.1. Background Knowledge of SAE

Deep learning (DL) algorithms have attracted immense attention owing to their excellent performances in numerous applications. SAE is an important deep learning algorithm and has gained wide applications due to its simply interpretability and excellent performances [33,34,35]. SAE is constructed with a few AEs and is trained using the layer-by-layer strategy [36,37,38]. Here we present the background knowledge of both AE and SAE.

**Figure 1:**
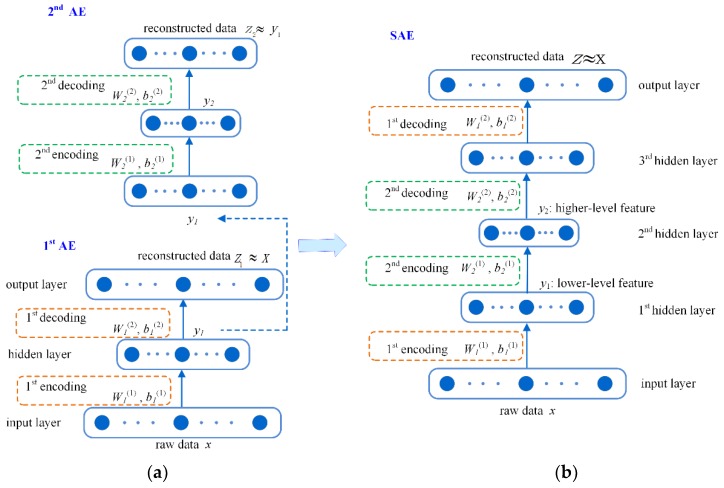
(**a**) Autoencoder (AE) and (**b**) stacked Autoencoder (SAE).

AE is a specific neural network (NN) model with one hidden layer as indicated in Figure 1a. AE is trained to guarantee the model output to be as close as possible to its input, which is different from the training objective of a common NN [39,40]. AE works through two steps: encoding and decoding. Encoding maps the input of the model to the output of the hidden layer through:

(1) $$y=f(W^{(1)\;}x+B^{\left(1\right)})$$

and decoding maps the output of the hidden layer to that of the output layer through

(2) $$z=g(W^{(2)\;}x+B^{\left(2\right)})$$

where *W*^(1)^, *W*^(2)^ are the weight matrixes between two adjacent layers. *B*^(1)^ ∈ *R^h^*, *B*^(2)^ ∈ *R^N^* are the bias vectors, where *h* and *N* are the number of hidden layer neurons and that of the output layers. *f*(•) and *g*(•) are the activation functions of the hidden layer and the output layer, respectively. The parameters of one AE model are learned by minimizing the special cost function as follows:

(3) $$\left(W^{\left(1\right)},W^{\left(2\right)},B^{\left(1\right)},B^{\left(2\right)}\right)=\underset{W^{\left(1\right)},W^{\left(2\right)},B^{\left(1\right)},B^{\left(2\right)}}{\mathrm{argmin}}\left(\sum_{i=1}^{m}{∥z^{\left(i\right)}-x^{\left(i\right)}∥}^{2}/2m+\lambda \sum W^{2}/2\right)$$

where *m* is the quantity of the training samples. The first term is the sum-of-square difference between the input and the output of the model. The second term provides additional suppression of the weight magnitude to prevent overfitting. *λ* is the weight decay parameter, which is a hyperparameter controlling the effect of the second term on the model. This minimization problem is solved using backpropagation algorithm which is the conventional approach for training a NN model. The details about backpropagation algorithm can be found in [41].

After an AE model is trained so its output is as close as possible to its input, the input data can be reconstructed at the output layer through the hidden layer using the trained model. Consequently, important information contained in the input data remains in the output of the hidden layer. Hence, the output of the hidden layer is an effective representation of the input data. Moreover, by constraining the neuron quantity in the hidden layer smaller than that in the input layer, we reduce the data dimension and obtain a compact representation of the raw data. In this compact representation, the noise is usually effectively eliminated [37].

SAE is a deep learning algorithm which has a deep architecture constructed with several AEs [42]. Figure 1b illustrates a five-layer SAE constructed with two AEs shown in Figure 1a. The parameters (*W*_1_^(1)^, *b*_1_^(1)^, *W*_1_^(2)^ and *b*_1_^(2)^) enclosed with the yellow boxes correspond to the first AE. The parameters of the second AE are denoted as *W*_2_^(1)^, *b*_2_^(1)^, *W*_2_^(2)^ and *b*_2_^(2)^ and are enclosed with the green boxes. SAE is trained for the same objective as AE, i.e., its final output should be as close as possible to its input.

A SAE model can be effectively trained by employing the layer-by-layer greedy training procedure, which is the normal training strategy in DL literature. This training procedure is illustrated in Figure 1. Firstly, the 1st AE is trained using the backpropagation algorithm to make its output (*z*_1_) being as close as possible to its input data (*x*). Then, the hidden layer output of this trained AE (*y*_1_) is employed as the input to train the 2nd AE. After both AEs have been trained, the learned parameters are employed as the initial values of the SAE model as illustrated in Figure 1b. Then a fine-tuning procedure is implemented to optimize the SAE model. This fine-tuning procedure aims to further decrease the difference between the output and the input of the SAE model. This procedure is also achieved by using the backpropagation algorithm. After fine-tuning, the SAE model provides several alternative representations at multiple levels for the input data. Representation at each level is one extracted feature in the input data. The output of the 1st hidden layer is an alternative representation describing the lower-level feature in the input data. That of the 2nd hidden layer describes the higher-level feature of the input data. In this study, we use the output of the 2nd hidden layer to construct new images for better indicating delaminations.

### 2.2. The Proposed SAE-PT Approach

#### 2.2.1. Preprocessing Thermograms

**Figure 2:**
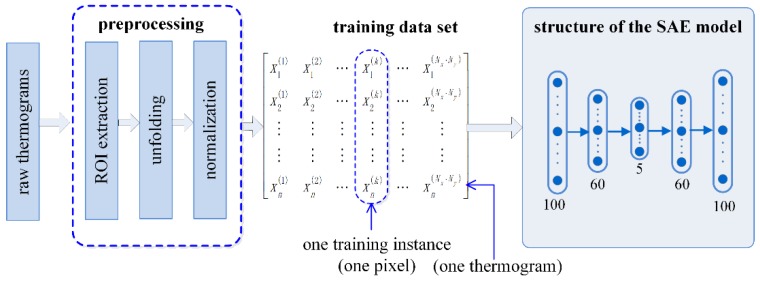
Training data set for the SAE model and the SAE structure used in this study.

In PT inspection, raw inspection data is a sequence of images (termed thermograms in the literature) captured by an IR imager. These images provide the training samples for the proposed approach. Before being used as training samples, the raw inspection data is first preprocessed through three steps, including region of interest (ROI) extraction, unfolding and normalization (Figure 2).

In PT inspection, each thermogram indicates the temperature distribution within the inspection scope at a specific moment. Usually both the inspected specimen and other ambient background are presented in the thermograms. The first preprocessing step ROI extraction aims to remove the ambient background from the raw thermograms and only remain the inspected specimen. During PT inspection, the inspected specimen always has much higher temperature than the ambient background. Hence, ROI extraction can usually be achieved by using a simple thresholding method. With using *N*_x_ and *N*_y_ to denote the width and the length of the ROI and using *n* to represent the length of the involved thermogram sequence, the data obtained by ROI extraction can be represented by a 3D matrix with dimension of *N*_x_ × *N*_y_ × *n*.

Then, an unfolding procedure is implemented to transform the 3D matrix to a 2D one with dimension of *n* × (*N*_x_ × *N*_y_). In the 2D unfolded matrix, each column corresponds to the temperature evolution of one pixel while each row corresponds to one thermogram frame.

This unfolded matrix is then normalized so that each column has a zero-mean and each element has a value in [0.1, 0.9]. This normalization is achieved by subtracting the mean value of each column from the original element of this column. Then the data out of +/−3 standard deviation (*σ*) are regarded as outliers and removed. The data larger than 3σ are replaced by 3σ while those less than −3*σ* are replaced by −3*σ*. This truncation processing is helpful for reducing noise in the raw data to some extent. Lastly, the truncated data is linearly transformed into the scale of [0.1, 0.9].

After the above described preprocessing, the raw inspection data obtained through one PT inspection is transformed into a 2D normalized matrix with dimension of *n* × (*N*_x_ × *N*_y_). Each column of this matrix corresponds to one pixel while each row corresponds to one thermogram. This 2D matrix provides all input instances for the SAE model. This matrix is first used to train the SAE model and then to construct the SAE images using the trained model.

#### 2.2.2. Training SAE Model

In this study, we preliminarily employ a five-layer SAE to investigate the potential of SAE to enhance delamination visibility during PT of CFRP. The five-layer SAE model is constructed with two AE models, as previously shown in Figure 1b. The detailed structure of the SAE model is demonstrated in the right part of Figure 2.

In a SAE model, the neuron quantity of the input layer is determined by the dimension of one input instance. When using SAE to process natural images, pixel values in an image or in an image patch are commonly used as an input instance of the SAE model. In this study, we process thermogram sequence in a different way. The temperature evolution of each pixel other than each thermogram is used as one input instance. Hence, the neuron quantity of the input layer equals to the length of the involved thermograms, which is set as 100 in this study. Meanwhile, the quantity of training instances equals to the pixel number in the ROI. For example, if the ROI contains 250 × 320 pixels, then the model is trained with 8 × 10^4^ training instances.

As stated previously, a compact alternative representation of the raw data can efficiently decrease the noise level. Therefore, we constrain the neuron quantity in the 1st and the 2nd hidden layer to be less than that of its previous layer. We choose 60 and 5 as the neuron quantity for these two layers, respectively. As a result, the constructed SAE model has an architecture of 100 × 60 × 5 × 60 × 100 as shown in Figure 2.

This SAE model is trained using the training data set illustrated in Figure 2. Concretely, after one inspection, the raw inspection data is preprocessed to a 2D matrix through the steps described in Section 2.2.1. Each column of the 2D matrix is then fed into the model as one training instance. By employing the layer-by-layer strategy and the fine-tuning procedure, which are described in Section 2.1, a specific SAE model is trained for the inspection. During the training procedure, the number of iterations for each AE is set as 100. When fine-tuning the SAE model, we set the learning rate as 0.3 and the number of iterations as 10 in this study.

#### 2.2.3. Constructing SAE Images

**Figure 3:**
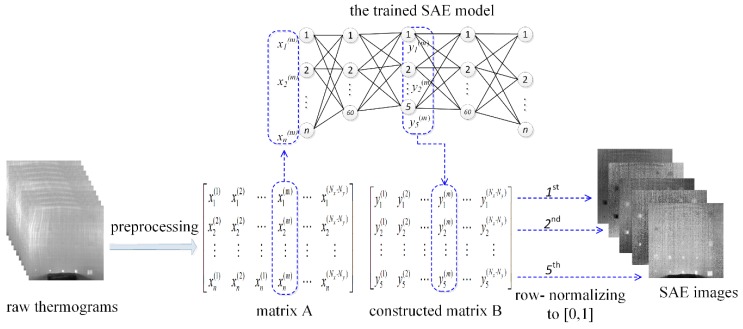
Construction of the SAE images.

Using the SAE model specifically trained for one inspection, we construct SAE images for this inspection. Figure 3 illustrates the procedure for constructing the SAE images. The matrix A obtained through preprocessing the raw inspection data, which has been used as the training data set, is again employed here to construct the SAE images. Each column of this matrix A, which is the normalized temperature evolution of each pixel, is sequentially fed into the input layer of the trained model. Then, the trained SAE model computes the lower-level feature of the temperature evolution for each pixel at the output of the 1st hidden layer. The higher-level feature of the raw data is obtained at the 2nd hidden layer. In the proposed approach, this higher-level feature of the raw inspection data is used to construct new images, which are expected to reveal the delaminations more clearly.

By sequentially feeding each column of matrix A into the trained SAE model, we obtain the output of the 2nd hidden layer for each pixel. The output of the 2nd hidden layer for all pixels are then organized as a new matrix B, each column of which corresponds to one pixel as shown in Figure 3. Then matrix B is row-normalized through linearly scaling values in each row to [0, 1]. At last, we fold each row of the normalized matrix to construct a new image. The folding is implemented as an inverse operation of the unfolding in the preprocessing stage. As a result, each row of matrix constructs one new image. Since the 2nd hidden layer has five neurons in this study, matrix has five rows then five new images are obtained for one inspection.

## 3. Experiments

To verify the effectiveness of the proposed SAE-PT, we implemented PT inspections on two CFRP specimens with prefabricated delaminations. The information of the specimens and the experimental setup are described in this section.

**Figure 4:**
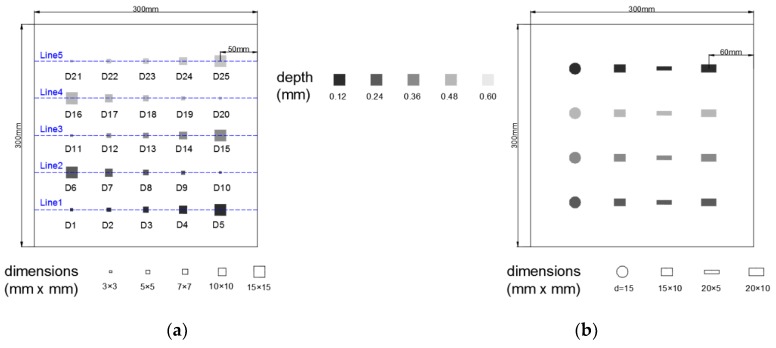
Sketch of the specimen A (**a**) and the specimen B (**b**). The dash lines used in the discussion section are also demonstrated in (**a**).

In this study, the two specimens under inspection were CFRP plates with multiple prefabricated delaminations, as shown in Figure 4. The specimens were manufactured with 15 layers of pre-impregnated satin woven carbon fiber cloth which is fabricated with TAIRYFIL-TC35/12k (Formosa Plastics Corporation, TaiWan) and epoxy resin. The final thickness of the specimens is 1.5 mm. Each prepreg layer was first sized as 300 mm × 300 mm and then stacked in the 0°/90° orientation. Between specified adjacent layers, pieces of polytetrafluoroethylene membranes (PTFE) with thickness of 0.1 mm were laid to simulate delaminations. The assembly was then placed into a hot-press that was preheated to 50 °C and pressurized to 8 MPa. The temperature in the hot-press was increased every 15 min at 15 °C increments until reaching 80 °C. Then, the laminate was cured for an additional 3.5 h at 80 °C followed by a cooling procedure of 12 h at room temperature.

Specimen A is used to evaluate the efficacy of the proposed approach in a detailed way. Specimen B is then used to test the generality of the approach. In specimen A, square PTFE of different sizes were arranged in different depths as described in Figure 4a. In specimen B, circular and rectangular delaminations were simulated at different depths as shown in Figure 4b.

**Figure 5:**
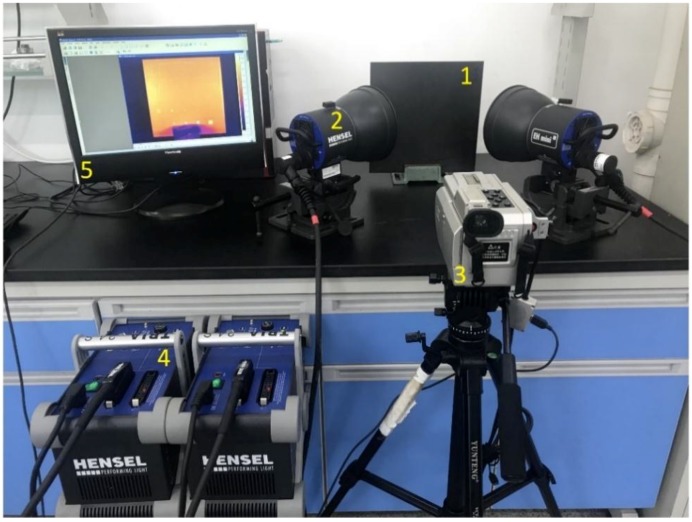
PT inspection equipment. 1-Specimen; 2-flash lamp; 3-infrared thermal imager; 4-high power generator; 5-computer.

The PT inspection system used in this study consists of two high-power generators (TRIA 24 S, Hensel-Visit GmbH & Co. KG, Germany), two flash lamps (EH MINI i, Hensel-Visit GmbH & Co. KG, Germany), an infrared thermal imager SAT-HY6850 (SAT Infrared Technology Co., Ltd, Guangzhou, China) and a computer. The photograph of the experimental setup is shown in Figure 5. High-power generators provide the energy to the flash lamps. Two flash lamps work synchronously and in total provide 4.8 kJ of power for 15 ms, 2 ms at full width half maximum as a high-power short pulse to heat the specimen. The infrared thermal imager works using polycrystalline silicon detectors which are sensitive to infrared wave with spectral range of 8–14 µm. The full spatial resolution of the thermal imager is 320 × 240 pixels and the temperature resolution is 80 mK. Considering the low heat conductivity of CFRP, we set the recording frequency of the imager as 25 Hz in this study. The temperature distribution of the specimen surface was recorded for 4 s and finally one hundred thermograms were captured.

## 4. Results and Discussions

### 4.1. Raw Thermograms and SAE Images

**Figure 6:**
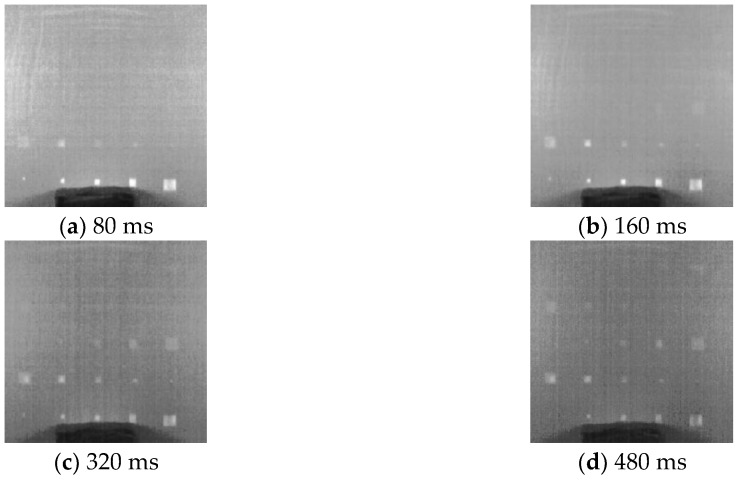
Raw thermograms.

Since the delaminations are located at different depths in specimen A, the most distinct indications occur in different thermograms for these delaminations. The clearest thermograms for each row delaminations are shown in Figure 6. These thermograms were captured at 80, 160, 320 and 480 ms after the flashing, respectively. From Figure 6a, we can observe that the shallowest delaminations at the 5th row in this specimen, have the most distinct indications. These distinct indications provide enough supports for inspectors to identify these defects. Figure 6b shows that the delaminations in the 4th row is indicated more blurrily compared to those in the 5th row. For the delaminations in the middle row, D13-D15 are indicated with low contrasts in Figure 6c. D12 is indicated with comparatively low contrast and can hardly be observed. D11 has no indications and cannot be observed at all in the raw thermogram. For the delaminations in the 2nd row, only D16-D18 have extremely weak indications in Figure 6d, which can be easily missed in the inspection. Even if inspectors notice the weak indications of these two defects, they cannot confidently identify them. Additionally, D19 and D20, and all defects in the 1st row cannot be observed from these raw thermograms.

**Figure 7:**
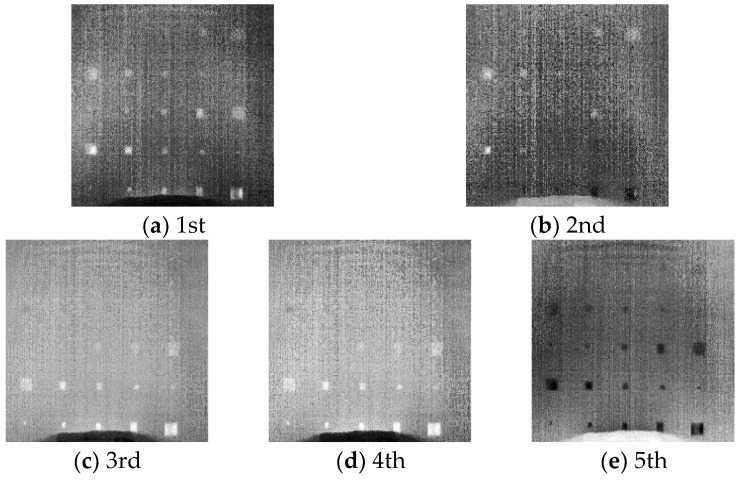
SAE images obtained through the proposed SAE–PT approach.

Using the developed approach described in Section 2, we preprocessed the raw detection data and then used the preprocessed data to train a SAE model. By feeding the preprocessed data into the trained SAE model, we obtained the higher-level representations of the raw inspection data in the second hidden layer output. We reorganized these higher-level representations and constructed five SAE images, as described in Section 2. The SAE images obtained based on the experimental data are shown in Figure 7. We can find that the 1st SAE image indicates delaminations as bright regions. Most delaminations can be observed in this image. The 2nd SAE image shows deep delaminations while both the 3rd and the 4th images indicates the shallow ones. The 5th SAE image is similar to the 1st one but shows delaminations as dark regions. By comparing the details in the 1st SAE image and those in the 5th one, we find that the 1st SAE image provides higher contrasts between defects and sound regions. Hence, the 1st SAE image is the best one in the SAE-PT results. In this SAE image, for the shallow delaminations in the bottom two rows, although they can also be identified from the raw thermograms, they are indicated as brighter regions. Meanwhile, the visibilities of the deep delaminations in the upper three rows are evidently enhanced comparing to those in the raw thermograms. For D13–D15 in the 3rd row, the contrasts of these defects are significantly improved in 1st SAE image. D11 and D12, two defects that can hardly be observed in the raw thermograms, can be noticed with an obviously higher visibility in the SAE image. The visibility of the delaminations in the 2nd row is also noticeably enhanced. D19, which has no indication at all in the raw thermograms, presents with a weak but identifiable indication in the SAE image. We can also observe that the right two delaminations at the 1st row (D24 and D25) become identifiable in the SAE image. Consequently, visibilities of most defects are enhanced in the SAE image. Enhanced defect visibility makes more delaminations identifiable in the SAE image than in the raw thermograms. In general, with the inspection equipment used in this study, the proposed approach can detect delaminations not smaller than 3 mm within the depth of 0.36 mm. Within the depth of 0.48 mm, the approach can detect delaminations not smaller than 5 mm. Within the depth of 0.6 mm, delaminations not smaller than 10 mm can be detected by SAE-PT using the same equipment.

**Figure 8:**
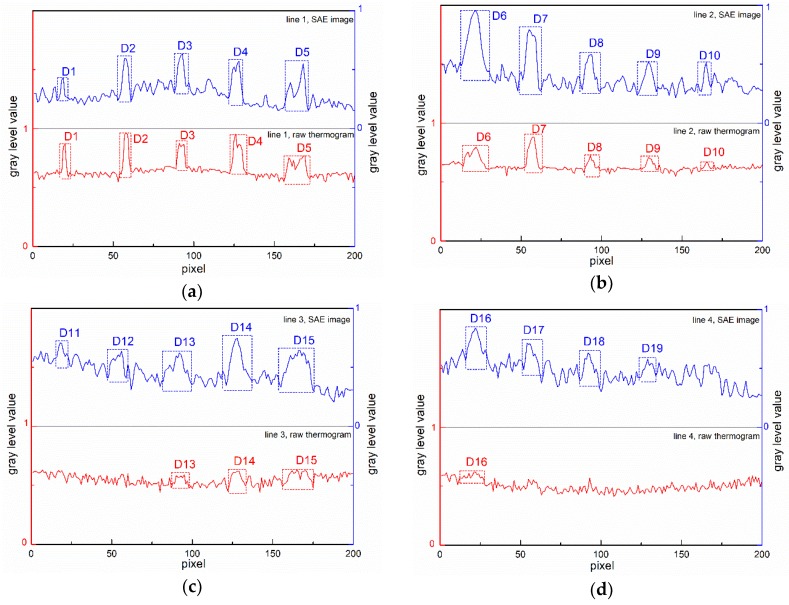

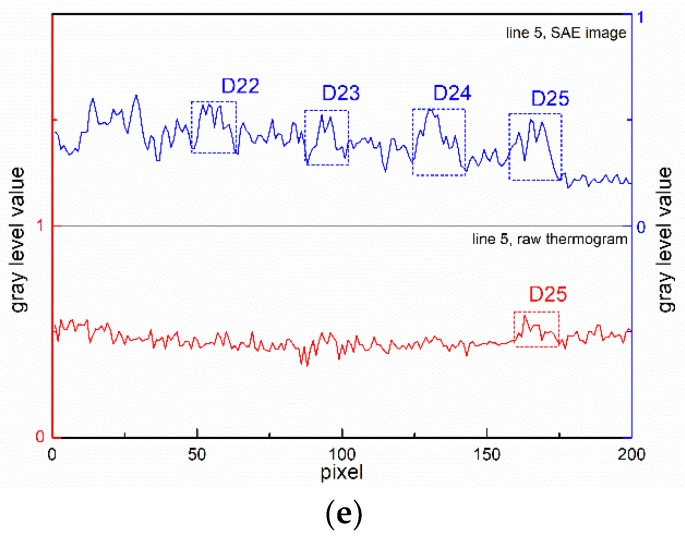
Gray level value variations in the raw thermograms and in the 1st SAE image along the investigation (**a**) line 1, (**b**) line 2, (**c**) line 3, (**d**) line 4, (**e**) line 5 shown in Figure 4.

Figure 8 illustrates the gray level variation in the raw thermograms and in the 1st SAE image, along the horizontal lines shown in Figure 4. The curves in blue describe the gray level variations in the SAE image while the red ones correspond to the raw thermograms. All identifiable delamination indications are marked with dashed rectangles. 

From Figure 8a, we can observe that for the delaminations in the 5th row (depth of 0.12 mm), the SAE image provides similar indications to the raw thermogram. For the 4th row delaminations (with depth of 0.24 mm), the SAE-PT approach evidently improves the identifiablility of each delamination as shown in Figure 8b. In Figure 8c, the anomalies result from delaminations become very weak in the raw thermogram. Only D13–D15 can be ambiguously identified from the variation curve of the raw thermogram. After implementing the SAE–PT approach, we can definitely identify D11-D15 from the variation curve of the SAE image. For the delaminations deeper than 0.4mm (in the 2nd row and the 1st row), only the largest delaminations (15 mm × 15 mm) show considerably weak indications in the variation curves of the raw thermograms. By contrast, the variation curve of the SAE image provides enough indications for four delaminations except the smallest ones.

### 4.2. Visual Comparison with PCT Algorithm

To further evaluate the developed SAE-PT approach, we compared it with the PCT method. PCT is an effective technique to enhance defect visibility during PT and has been widely employed in PT inspections.

**Figure 9:**
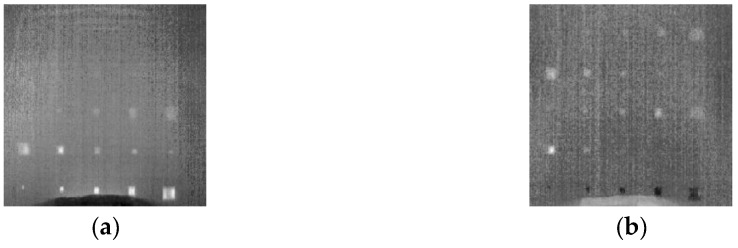
The EOFs obtained through PCT. (**a**) The 2nd EOF; (**b**) the 3rd EOF.

Figure 9 shows the 2nd and the 3rd empirical orthogonal functions (EOFs), which correspond to the 2nd and the 3rd principle components, respectively. From Figure 9a, we can observe that the 2nd EOF can reveal D1-D10 and D13-D15, however the other delaminations cannot be recognized from this EOF. The 3rd EOF reveals D13-D18 and D23-25, however some shallow delaminations (D8-D10) are lost in this image. Moreover, the indications of most delaminations are generally weak in this image. In comparison, the SAE image shown in Figure 7 indicates the presence of D1-D19 and D22-D25 in a more distinct manner. In the SAE image, most delaminations are easier to be noticed than in the EOFs. Especially, the delamination D8-D15 are shown brighter in the SAE image than in the 2nd and in the 3rd EOFs. Therefore, in term of enhancing the delamination visibility in CFRP, the proposed approach shows better performance than PCT, qualitatively.

It should also be noted that the EOFs show a better performance in weakening the effect of non-uniform heating. The non-uniform heating effect is not evident in either the 2nd EOF or in the 3rd one. However, this negative effect can still be observed in the SAE image. This is the reason why D16 is not so obvious in the SAE image although it has been shown as a brighter region than that in the EOFs. Consequently, compared with PCT, the proposed approach SAE-PT shows better performance in enhancing the visibility of delamination in CFRP. However, its ability to suppress the effect of non-uniform heating is not good as PCT.

### 4.3. Quantitative Evaluation of SAE-PT

Contrast is a conventional metric to evaluate the saliency of an object in a scene [43,44,45,46,47]. An object with a higher contrast to its surrounding background is easier to notice. As a result, here we use the contrast of a delamination to its surrounding region to evaluate its visibility in an image. Here we compare the delamination visibility in the SAE images to that in the raw thermograms, the 2nd and the 3rd EOFs, respectively.

**Figure 10:**
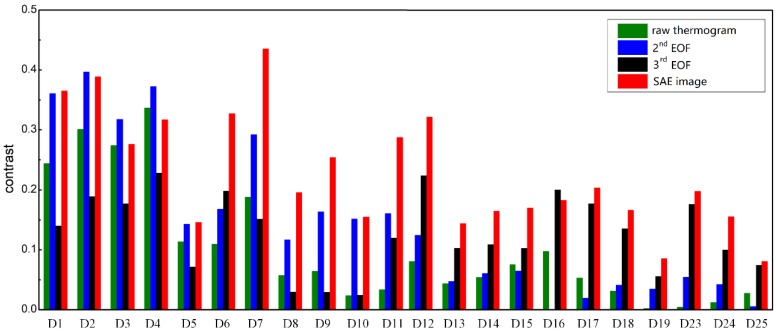
The calculated delamination contrasts of the raw thermogram, the EOFs and the SAE image.

Delamination contrasts are calculated as the intensity difference between a delamination region and its surrounding region. In this study, we first manually marked the delamination regions based on both visual perception. Then 10 × 10-pixel regions adjacent to each delamination from the right side were assigned as sound regions. The average gray level value of the assigned sound region represents the intensity of this region. The average value of the marked delamination region describes the intensity of the defect region. Then the difference between these two average values is calculated as the delamination contrast. The calculated delamination contrasts are illustrated in Figure 10.

From Figure 10, we can observe that the proposed approach generally provides higher delamination contrasts for most delaminations. For D2 to D4, which have been indicated clearly in the raw thermograms, the delamination contrasts in the SAE image are a little lower than those in the 2nd EOF. For D16, the 3rd EOF indicates this delamination with the highest contrast. However, for the other delamintions (D1, D5-D15 and D17-D23), SAE-PT provides the highest contrasts for defect indications compared to the raw inspection and PCT.

These comparison results demonstrate that the developed SAE-PT method is effective in increasing the delamination contrasts and correspondingly enhancing the delamination visibilities. By enhancing the delamination visibility, SAE-PT exhibits its potential to improve the inspection accuracy of PT for quality monitoring of CFRP.

### 4.4. Generalization of SAE-PT

**Figure 11:**
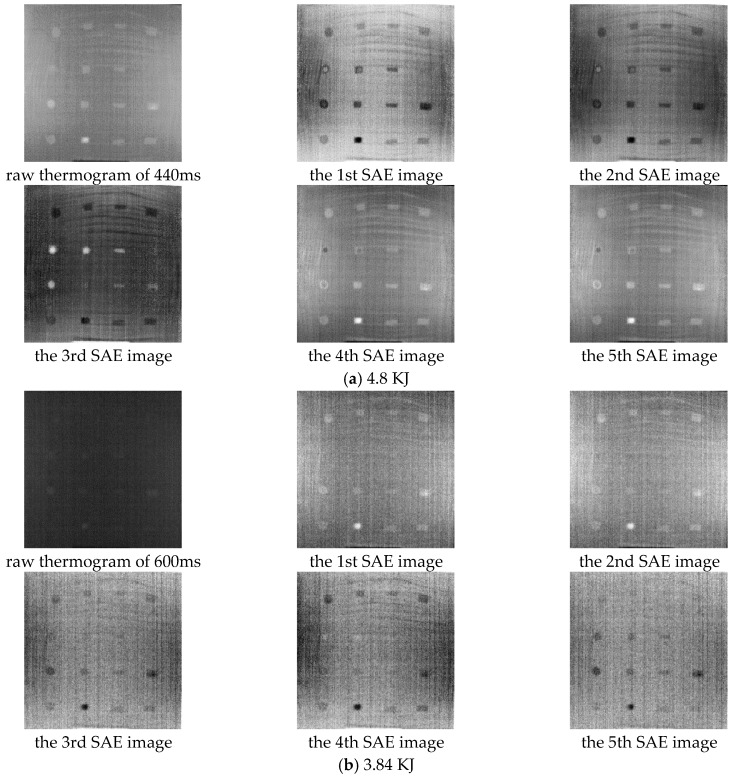
Raw thermogram and SAE images of specimen B. Flash heating energy: (**a**) 4.8 KJ, (**b**) 3.84 KJ.

To investigate the generalization of the proposed approach, we tested the approach on specimen B, in which there are circular delaminations and rectangular ones. The specimen was inspected twice, during which the flash heating is 3.84 and 4.8 KJ, respectively. The results of SAE-PT and a raw thermogram are listed in Figure 11. The upper left images in the Figure 11a,b is the raw thermograms captured at 0.44 s with 4.8 KJ and at 0.6 s with 3.84 KJ. These two thermograms are generally the best one in the raw thermogram sequence. By comparing the raw thermograms and the results of SAE-PT in Figure 11a, we can find that SAE-PT can effectively improve the delamination visibility and can indicate the shape of delaminations more clearly. Through Figure 11b, we can observe that most delaminations are not visible in the raw thermogram. After using SAE-PT, most delaminations except the rightmost one in the 2nd row can be identified through the SAE images. The delaminations in the 1st, 3rd, 4th row can be observed in the first four SAE images while three delaminations in the 2nd row are shown in the last SAE image. Moreover, the shape of these identifiable delaminations can be basically retrieved through the results of SAE-PT. Considering the great difference between the cost of a low-power flash heating equipment and that of a high-power one, SAE-PT provide a strategy to reduce the cost of PT inspection. By employing SAE-PT, a low-power flash heating PT equipment is expected to achieve similar inspection accuracy as a high-power one.

## 5. Conclusions

Enhancing the defect visibility is beneficial to improve the detection accuracy of pulsed thermography (PT). In this study, we integrate a deep learning algorithm called Stacked AutoEncode (SAE) with PT to develop a novel approach SAE-PT to enhance the delamination visibility during PT of carbon fiber reinforced plastics (CFRPs). In the proposed approach, higher level features are extracted from the raw thermograms and are employed to construct new images. Experiments on a CFRP plate with prefabricated delaminations demonstrate the efficacy of SAE-PT. We compare the delamination visibility of the reconstructed image of SAE-PT with those of the raw thermograms and the reconstructed image of Principal Component Thermography (PCT). The comparative study shows that SAE-PT provides higher contrasts for most delaminations, effectively enhancing the delamination visibility. This study demonstrates that deep learning algorithm is a powerful tool to improve the inspection capability of PT as it has performed in other areas. In the future, we will extend our effort to explore the potential of other deep learning algorithms to improve the inspection capability of infrared thermography.
